# The effect of exercise interventions on inflammatory markers in major depressive disorder: protocol for a systematic review and meta-analysis

**DOI:** 10.12688/hrbopenres.13240.1

**Published:** 2021-05-04

**Authors:** Diana Grunberg, Jason A. Martin, John F. Cryan, Ken D. O’Halloran, Eric Kelleher, Timothy G. Dinan, Gerard Clarke

**Affiliations:** 1 School of Medicine Brookfield Health Sciences Complex, University College Cork, Cork, Cork, T12 YT20, Ireland; 2 Department of Psychiatry and Neurobehavioural Science, University College Cork,, Cork, Cork, T12 YT20, Ireland; 3 APC Microbiome Ireland, University College Cork, Cork, Cork, T12 YT20, Ireland; 4 Department of Anatomy and Neuroscience, University College Cork, Cork, Cork, T12 YT20, Ireland; 5 Department of Physiology, University College Cork, Cork, Cork, T12 YT20, Ireland; 6 Department of Psychiatry and Neurobehavioural Science, Cork University Hospital and Mercy University Hospital, Cork, Cork, T12 YT20, Ireland; 7 INFANT Research Centre, University College Cork, Cork, Cork, T12 YT20, Ireland

**Keywords:** major depressive disorder; MDD; depression; inflammatory biomarkers; inflammatory cytokines; exercise;

## Abstract

**Background:** Depression currently affects 4.4% of the global population, and 93.7% of this population suffer from major depressive disorder (MDD) according to 2017 statistics. MDD patients are more likely to suffer from co-morbidities such as cardiovascular disease and high body mass index (BMI), thus contributing to its large cost to society. Throughout the literature, there are known links between inflammation and MDD. Interestingly, while exercise is considered a promising intervention for MDD, the mechanism(s) of action remain unclear, thereby preventing the creation of optimal, cost-saving, exercise “prescriptions” for those with MDD. Thus, the aim of this review and meta-analysis is to summarize and analyse the current literature exploring how quantified exercise interventions modulate inflammatory molecules in MDD patients.

**Methods:** Electronic databases (APA PsycINFO, and PubMed/MEDLINE (EBSCO interface), EMBASE) will be searched using a detailed search strategy comprised of three search term themes: exercise, depression/MDD, and inflammation/inflammatory molecules. Only quantified exercise interventions performed in adult humans with MDD validated via a recognized diagnostic criterion will be included. Studies should also include a MDD control group and explore changes in inflammatory molecules. Examples of these molecules include: C-reactive protein (CRP), interleukin-6 (IL-6), interleukin-1 beta (IL-1b), tumour necrosis factor-alpha (TNF-a), homocysteine, d-dimer and myeloperoxidase (MPO). After eligible studies are identified, standardized data extraction will be employed and the risk of bias in each study will be appraised using the Cochrane handbook checklists. In the event of two or more homogenous studies exploring exercise effects over a similar period of time, raw mean differences or standardized mean differences will be pooled using random effects analysis. This systematic review and meta-analysis will be reported according to the Preferred Reporting Items for Systematic reviews and Meta-Analyses (PRISMA) guidelines.

**Dissemination:** This systematic review and meta-analysis will be disseminated in peer-reviewed journals.

**PROSPERO registration: **CRD42020186006 (31/08/2020)

## Introduction

### Rationale

Depression is a leading cause of disability worldwide, affecting more than 300 million people or 4.4% of the global population today ^1^. In 2015, it was calculated that depressive disorders account for more than 50 million ‘years lived with disability’ (YLD) internationally, with the World Health Organization predicting that depressive disorders will be the leading cause of global health burden by 2030 ^1^. According to the Global Burden of Disease (GBD), the number of incident cases of depression worldwide has seen a steep increase from 172 million in 1990 to more than 258 million in 2017, whereby a large majority (93.7%) of these patients with depression suffer from major depressive disorder (MDD) ^2^. The Diagnostic and Statistical Manual of Mental Disorders, Fifth Edition (DSM-5), classifies MDD as a clinical depression syndrome where a patient experiences persistent low mood for at least two weeks, inclusive of other depressive symptoms: dysphoria, reduced motivation, psychomotor and cognitive dysfunctions (‘pseudo-dementia’), anhedonia, sleep and weight changes, and reduced libido ^3^. MDD is also associated with physical health-comorbidities—cardiovascular disease, high BMI, and premature mortality— all of which compound its high financial cost to society ^4^.

The connection between inflammation and MDD is well-recognized throughout the literature ^5– 9^. The mechanism of this relationship is described by the inflammation/cytokine model of depression, which purports that a pro-inflammatory state caused by an increase in pro-inflammatory cytokines and/or the reduction in anti-inflammatory cytokines results in the development of clinical depression in humans ^10– 12^. The pro-inflammatory cytokines involved in the cycle include tumour necrosis factor (TNF)-α, interferon (IFN)-γ, interleukin-1 beta (IL-1β), and interleukin-6 (IL-6), which is also known to have anti-inflammatory properties ^13^. The key cytokines that have been implicated in the sickness behaviours that overlap with MDD—anhedonia, social withdrawing, decreased activity—are TNF-α and IL-1β ^10^. IL-6 is less strongly associated, although it is elevated in those with SSRI-resistant depression ^14^. Interestingly, TNF-α and IL-6 levels were found to be particularly elevated in depressed subjects compared to controls in a 24 study meta-analysis conducted by Dowlati *et al.* ^15^.

It has been consistently reported throughout the literature that exercise has antidepressant effects ^16^. For example, a meta-analysis of 49 prospective cohort studies (1,837,794 person-years) by Schuch *et al.* determined that participants with higher exercise frequency had a 17% reduction in odds of developing depression ^16^. On the other hand, there is evidence that low cardiorespiratory fitness (CRF), an indicator of physical inactivity, yields a 64% increased risk of developing depression compared to those with high CRF across at least 3,540,450 person-years of data ^17^. Apart from these findings, exercise has also been shown to enhance self-esteem and has less stigmatization than psychotherapy ^18^. The low side-effect profile and adaptation opportunities of exercise based on a patient’s comorbidities makes it an attractive intervention when considering the complexity of depression in relation to pharmacological and psychotherapeutic options ^19^. Despite the apparent utility of exercise in treating MDD, the precise mechanisms and mediators underlying its antidepressant effects remain to be elucidated, thus prohibiting the creation of optimal exercise treatments for MDD patients.

It is known that IL-6 levels are increased post-exercise when it is released from skeletal muscles, and it has been shown to inhibit the production of TNF-α; as such, IL-6 may be anti-inflammatory in the context of immediate exercise ^20^. However, the mediation of IL-6 in relation to its pro-inflammatory function, along with other similar molecules, is still being explored with reference to different exercise styles and durations. According to a longitudinal exercise study by Kohut *et al.*, cardiovascular exercise performed three times per week for 45 minutes per day over 10 months resulted in decreased serum levels of CRP and IL-6 ^21^. It was also found that reductions in TNF-α were seen in both the cardiovascular group and flex group, who performed elements of yoga, Tai Chi, flex band, free hand weights and stability balls over the same time period ^21^. Interestingly, a shorter-term exercise 10-week study by Dongers *et al.* showed that resistance exercise training reduced CRP levels by 32.8%, compared to the aerobic group which only saw a 16.1% reduction ^22^. Therefore, there is evidence from exercise training research that suggests the long-term anti-inflammatory outcome of exercise could be due to the reduction of pro-inflammatory molecules.

Considering the (1) inflammatory aetiology of MDD, the (2) connections between inflammation and exercise, and the (3) urgency for accessible economical treatments for MDD on a global scale, it would be useful to both the scientific and medical community to perform a novel systematic review and meta-analysis that will inform areas for further research and identify gaps in the field. It is important to note that this study will only evaluate the impact of quantified exercise interventions, rather than “physical activity” broadly. Such terms are often conflated, or used inter-changeably, in the literature despite their distinct definitions. Physical activity (PA) refers to any bodily movement by the skeletal muscles that necessitates energy expenditure (e.g. gardening, leisurely walking, sports); on the other hand, exercise is defined as a subset of PA that is structured, planned, and repetitive, and has the objective of improving or maintaining fitness ^23^. Thus, our inclusion of only quantified exercise interventions aims to ensure standardization across all studies in order to increase the validity of review findings.

### Objectives

The purpose of this research is to systematically review previous studies that have investigated the effects of quantified exercise interventions on inflammatory cytokine and biomarker levels in individuals diagnosed with MDD, via validated criteria, as compared to controls without any other major psychiatric or somatic co-morbidities.

The second focus of this review is to undertake a meta-analysis on inflammatory molecule changes pre- and post- intervention in MDD patients included in the systematic review studies.

Further objectives include:

(1) Evaluate the inter-study consistency of MDD populations and the criteria of diagnosis.

(2) Establish which inflammatory molecules are most studied in the MDD populations in research that involves an exercise intervention.

(3) Evaluate different exercise modalities, the exercise intensity, exercise session duration, and exercise intervention duration (i.e., acute vs chronic), and how they modulate inflammation in MDD patients.

(4) Ascertain similar studies that measured the same outcomes, to determine those suitable for meta-analysis.

This protocol is reported in line with the Preferred Reporting Items for Systematic review and Meta-Analyses Protocols (PRISMA-P) guidelines ^24^.

## Methods

### Eligibility criteria

Studies will be selected for inclusion in the systematic review and meta-analysis according to the following criteria: 

***Study designs.*** We intend to include all published intervention studies examining the effects of an exercise intervention on inflammatory molecule levels in human MDD patients and controls participating in interventional exercise studies. Exercise intensity and duration must be reported. The following study designs will be excluded from this review and meta-analysis: systematic reviews, meta-analyses, case-studies, qualitative studies or quantitative studies performed on animal models or human populations with sub-depressive symptoms or those with psychiatric or somatic co-morbidities.

***Participants.*** The study population must be human, aged 18 years or older, with a clinical diagnosis of MDD according to a recognised, widely used diagnostic classification approach i.e. the Diagnostic and Statistical Manual for Mental Disorders (DSM), or International Classification of Diseases (ICD), or Research Diagnostic Criteria (RDC). Diagnosis should be confirmed with a structured interview using one or more of the following: Mini International Neuropsychiatric Interview (MINI), Composite International Diagnostic Interview (CIDI), Structured Clinical Interview (SCID), or equivalent. Depressive symptoms and severity should be scored using one or more standardized and validated scales. As example: Beck Depression Inventory (BDI), Hamilton Depression Rating Scale (HDRS/HAM-D), Centre for Epidemiologic Studies Depression Scale Revised (CESD-R), Montgomery-Åsberg Depression Rating Scale (MADRS), or equivalent.

***Interventions.*** The systematic review and meta-analysis will evaluate the impact of a quantified exercise intervention that should specify the following parameters: exercise (type, mode, frequency, and duration); work done (measures of power (watt) or energy transferred (joules)); measures of heart rate, repetitions performed; duration of each session; number of total sessions; duration of the intervention. Interventions evaluating yoga or tai-chi only, or those combined with pharmacological treatments, will not be included in this review.

***Comparators.*** Study control groups (i.e., not performing intervention) should include those diagnosed with MDD by the same standards as the participants and not having any other major psychiatric or health co-morbidities. Controls should ideally have the same amount of interaction time with the researchers.

***Outcomes.*** Studies reporting results for one or more of the following inflammatory cytokines and/or biomarkers will be selected. Examples of these molecules include: C-reactive protein (CRP), interleukin-6 (IL-6), interleukin-1 beta (IL-1β), tumour necrosis factor-alpha (TNF-a), homocysteine, d-dimer and myeloperoxidase (MPO).

***Time frame.*** There will be no restrictions on the length of follow up of outcomes.

***Setting.*** There will be no restrictions on type of setting for the interventions.

***Report characteristics.*** We will only include studies reported in the English language, due to limited resources to adequately translate studies. Only published journal articles available from our selected online databases will be included.

### Information sources

The following electronic databases will be searched: EMBASE, APA PsycINFO (EBSCO interface), PubMed/MEDLINE (EBSCO interface). The literature search will include all searchable published articles up until current day (2021). Literature saturation will be ensured by examining study references as well as identifying related systematic reviews for further relevant studies. While specific authors will not be contacted, research by prominent authors in the field will be screened to ensure all relevant material has been captured.

### Search strategy

Planned limits on the search include papers published in the English language only. The search strategy will include terms relating to the following three subject categories.

1. Depression: major depressive disorder, MDD, depressive episode
2. Inflammatory molecules: inflammation, biomarkers, inflammatory biomarker, inflammatory, inflammatory cytokines, cytokines, C-reactive protein, c-reactive protein, CRP, interleukins, interleukin-6, IL-6, interleukin-1 beta, IL-1B, IL-1beta, tumour necrosis factor-alpha, TNF-a, TNF-alpha, homocysteine, d-dimer, myeloperoxidase, MPO
3. Exercise intervention: exercise/exercising, physical activity, fitness, sedentary, daily activity, sport/sports, high intensity interval training, HIIT, cardiovascular exercise, cardiovascular training, cycle/cycling, bicycle/bicycling, exercise bicycle/bike, stationary bicycle/bike, ergometer, run/running, row/rowing.

Further search terms will be identified via descriptive terms under MeSH terms. The content of preceding search strategy content has been formulated and agreed upon by all authors of the study. A Health Sciences Librarian with expertise in formulating systematic review searches will be asked to help refine the search strategy for each database. The EBSCO interface search strategy, which can be used both for Pubmed/MEDLINE and PsychINFO databases, will be adapted for input into the EMBASE database interface. The detailed search strategy is presented as extended data ^24^.

### Data collection, extraction and assessment

***Study selection process.*** Two authors (DG and JM) will independently screen titles and abstracts for all articles, retrieved by the search, in order to identify studies that meet the inclusion and exclusion criteria. The full texts of all selected and potentially relevant articles will be collected and then independently examined by both authors to decide whether or not all eligibility criteria have been met at this phase. Any disagreements will be mediated through discussion and/or a third reviewer (GC). Duplicates will be excluded. A PRISMA flow chart will display the articles examined at each stage, detailing the number of papers included and excluded and reasons for exclusion. All reviewers will be unblinded to the journal titles, study authors and institutions.

***Data extraction and collection process.*** The following data items will be extracted from all studies and tabulated: study author and publication date, sample size (N), mean age and sex breakdown of participants (N and %), MDD diagnostic criteria, intervention type, intervention intensity, length of individual intervention sessions, duration of intervention (number of total sessions to discern acute/chronic exercise interventions), outcome(s) measured. Data items will be manually extracted independently by two authors (DG and JM). Software will be used to organize and compare data extracted by both reviewers. As in the selection process, any disagreements between reviewers (DG and JM) will be arbitrated through discussion and/or with the aid of the third author (GC). Any uncertainties regarding data will be resolved by contacting study authors via email.

***Outcome measures and prioritisation.*** The outcomes of interest included in this study fall into two broad categories: pro-inflammatory cytokines and inflammatory biomarkers. Cytokines are non-structural proteins that are produced by nearly all nucleated cells in the body, and they can be classified either as pro-inflammatory or anti-inflammatory ^25^. Pro-inflammatory cytokines function to induce inflammation by modulating gene expression; examples include interleukin-6 (IL-6), interleukin-1 beta (IL-1B), and tumour necrosis factor-alpha (TNF-a) ^25^. C-reactive protein (CRP), homocysteine, d-dimer and myeloperoxidase (MPO) are not categorized as pro-inflammatory cytokines but are known as inflammatory biomarkers; they are highly associated with inflammatory processes and have been found to either induce or mediate pro-inflammatory cytokines ^26– 29^.

In summary, our primary outcome measures of interest include those molecules most highly associated with pro-inflammatory processes. Examples of these molecules are C-reactive protein (CRP), interleukin-6 (IL-6), interleukin-1 beta (IL-1B), and tumour necrosis factor-alpha (TNF-a). Secondary outcome measures of interest include inflammatory biomarkers such as homocysteine, d-dimer and myeloperoxidase (MPO). It is important to note that throughout the study, pro-inflammatory cytokines and inflammatory biomarkers will be collectively referred to as inflammatory molecules.

### Risk of bias

Two review authors (DG and JM) will independently assess the quality of each study according to study design using existing appraisal checklists provided in the Cochrane handbook, including the Risk of Bias (RoB) 2 tool for randomised controlled trials ^30^. Meta-biases, such as outcome reporting bias, will be explored by evaluating whether or not the study’s protocol was published before the recruitment of participants; this will be performed for all available protocols. Trial registries will also be reviewed to establish if reported outcome measures and statistical methods reported in the studies match their original protocols.

Data quality for each study will be recorded in a spreadsheet and a table summarising the quality of assessment/evidence will be compiled and included in the systematic review and meta-analysis. The risk of confounds will be reduced by independently examining each study for potential confounds and how they were controlled for in the published study design (e.g., inclusion criteria selectivity) or analysis (e.g. employing adjustment techniques or analysis of covariance). To reduce the risk of bias in determining study quality, all discrepancies will be resolved via reviewer consensus or through consultation with a third reviewer (GC).

### Data synthesis

Studies will be grouped according to intervention type (i.e., acute or chronic) and summary tables of all characteristics of all articles included in the review compiled. Tables will outline the following: characteristics of study populations (N, age, sex), MDD diagnostic criteria, study design, intervention/test type, intensity, and outcomes. A narrative will summarise the findings of the aforementioned tables and explore inter- and intra-study relationships.

In the event of two or more homogenous studies in the same acute or chronic intervention group and having the same outcome unit of measure and comparator, the raw mean difference will be used in the meta-analyses (e.g. Beck Depression Inventory Score). Where studies use different measurement units, the raw mean difference (RMD) values will be extracted from each study and transformed to standardised mean difference (SMD) values to allow comparison between different units of measure. The RMDs or the SMDs, as appropriate, will be pooled using random-effects analysis and will be displayed in a forest plot with 95% confidence intervals (CIs) and weights. Cohen's criteria (Cohen 1998) will be used to interpret effect sizes: small (0.2), medium (0.5), and large effects (0.8). Subgroup analysis will be employed to explore sources of heterogeneity based on the following paraments: patient characteristics, MDD criteria used for diagnosis, intervention type (acute vs. chronic), intervention mode (for example, bike vs. ergometer). Analyses will be performed using SPSS. In the event of missing data, we will endeavour to contact the original study authors to obtain it. 

The Review Manager (Cochrane Collaboration Software, Rev-Man) software version 5 will be used to perform statistical analysis and to combine results in a forest plot, using random-effect models. The pooled OR and standard error (SE) will be used to generate a funnel plot in order to estimate the likelihood of publication bias if 10 or more studies are included in the meta-analysis.

### Confidence in cumulative evidence

The Grading of Recommendations Assessment, Development and Evaluation (GRADE) working group methodology will be employed to evaluate the quality of evidence. The following domains will be assessed for each PICO outcome: risk of bias in individual studies, inconsistency of results between studies, indirectness of evidence, imprecision, publication bias, and factors that increase the quality of evidence ^31^. Quality of evidence will be graded in terms of the level of confidence that the true effect lies close to that of the estimated effect (i.e., high, moderate, low and very low) ^31^.

### Ethics and dissemination

Ethical approval is not required for this study as it does not involve the inclusion or conduct of any experimental or personal data that would require informed consent. This systematic review and meta-analysis will be disseminated in peer-reviewed journals.

### Amendments

In the event of protocol amendments, the date of each amendment will be documented alongside a description of the change and its rationale. Amendments will also be recorded and tracked on the PROSPERO registry of this protocol.

### Study status

The study was first registered on PROSPERO (31/08/2020) with the protocol finalised in March 2021. The literature search will be completed in May, data extraction and risk of bias assessment will be completed in June, the statistical analysis will be completed in July/August and we expect to finish the study in September 2021.

Any protocol amendments will be stated in the review article. Any additional analyses will be reported in the review article and stated post-hoc.

## Discussion

By providing an up-to-date systematic review and meta-analysis, this study will synthesize current evidence of how exercise interventions modulate inflammatory molecule levels in those with diagnosed MDD compared to controls. Changes in pre- and post-intervention inflammatory molecule levels will be statistically summarised via meta-analysis, which will analyse acute and chronic exercise interventions, separately. The underlying goal of this study is to explore if exercise has anti-inflammatory effects in those with MDD, in relation to different types of activity and duration. These identified findings may indicate areas for further research and substantiate more targeted “exercise prescriptions” for those suffering from MDD.

### Potential limitations

This study may have several limitations. The search will be restricted to English language publications only. While not including unpublished literature possibly results in an increased risk of publication bias for the included studies, given that reports that describe a significant finding or a positive outcome have a greater probability of being published when compared to those that do not. In any case, a funnel plot will be used to evaluate the presence of publication bias. Studies focusing on adolescent or child populations will also be excluded from the data set, thereby limiting the number of potentially relevant research included in this review and meta-analysis. In addition, the inclusion of only validated MDD populations and specific inflammatory molecules may disregard other applicable studies. Sources of heterogeneity across the studies, in criteria such as patient characteristics, MDD criteria used for diagnosis, intervention duration type, and intervention mode, may also limit meta-analysis findings. If possible, subgroup analyses will be undertaken to evaluate the impact of these sources of heterogeneity.

## Data availability

### Underlying data

No data are associated with this article.

### Extended data

Open Science Framework: The effect of exercise interventions on inflammatory markers in Major Depressive Disorder. https://doi.org/10.17605/OSF.IO/DCQS5 ^24^

This project contains the following extended data:

- Web Interface Search Strategy.pdf (Study search strategy)

### Reporting guidelines

Open Science Framework: PRISMA-P checklist for ‘The effect of exercise interventions on inflammatory markers in Major Depressive Disorder’. https://doi.org/10.17605/OSF.IO/DCQS5 ^24^

Data are available under the terms of the Creative Commons Attribution 4.0 International (CC-By Attribution 4.0 International)
