# Supplementary material for: The effect of exercise interventions on inflammatory markers in major depressive disorder: protocol for a systematic review and meta-analysis
Source: HRB Open Res. 2022 Jun 15;4:42. Originally published 2021 May 4. [Version 3] doi: 10.12688/hrbopenres.13240.3 (PMC9549376; doi:10.12688/hrbopenres.13240.3)
Supplement: Peer review history

# Peer review: Reviewer response for version 3

**DOI:** 10.21956/hrbopenres.14832.r32329

**Published:** 2022-06-29

**Authors:** Felipe Barreto Schuch

**Affiliations:** 1 Department of Sports Methods and Techniques, Federal University of Santa Maria, Santa Maria, Brazil

I have no further questions!

Is the study design appropriate for the research question?

Yes

Is the rationale for, and objectives of, the study clearly described?

Yes

Are sufficient details of the methods provided to allow replication by others?

No

Are the datasets clearly presented in a useable and accessible format?

Not applicable

Reviewer Expertise:

NA

I confirm that I have read this submission and believe that I have an appropriate level of expertise to confirm that it is of an acceptable scientific standard.

# Peer review: Reviewer response for version 2

**DOI:** 10.21956/hrbopenres.14786.r31865

**Published:** 2022-05-04

**Authors:** Felipe Barreto Schuch

**Affiliations:** 1 Department of Sports Methods and Techniques, Federal University of Santa Maria, Santa Maria, Brazil

**The authors satisfactorily responded to my questions. However, the responses were not incorporated into the body of the manuscript.**

**For example:**

**When I referred to the 2016 citation, I suggested to the authors to strengthen the need and the novelty of their study, discussing the extant literature and showing how this new paper will further expand the literature. However, they have just added:** ***“*** *We are aware of a systematic review regarding the neurobiological effects of exercise on major depressive disorder, which provided evidence that exercise training had no significant acute or chronic adaptations on any inflammatory marker reviewed in people with MDD* *20* *.”*

**In the response letter, the authors respond:** *“We believe that a 6-year time gap is an appropriate timeframe within which to look anew on the data. The additional novelty of our review is its specific focus on how quantified exercise interventions modulate inflammatory biomarkers in MDD patients that have been published to date rather than the authors more expansive review that accounts the adaptations on neurogenesis, inflammation biomarkers and brain structure and how associations between improved depressive symptoms relate to hippocampal volume. We believe that our review will extend the observations around inflammation specifically beyond 2016 and is necessary to restate that the paucity of studies remains in this area of research and that limitations presented within existing studies precludes a definitive conclusion that exercise reduces inflammation in people with MDD.”*

**I agree entirely with the authors. This response is exactly what should be incorporated into the introduction**. 

 The same should be done for the responses to the other questions I've made.

Is the study design appropriate for the research question?

Yes

Is the rationale for, and objectives of, the study clearly described?

Yes

Are sufficient details of the methods provided to allow replication by others?

No

Are the datasets clearly presented in a useable and accessible format?

Not applicable

Reviewer Expertise:

NA

I confirm that I have read this submission and believe that I have an appropriate level of expertise to confirm that it is of an acceptable scientific standard, however I have significant reservations, as outlined above.

# Peer review: Reviewer response for version 2

**DOI:** 10.21956/hrbopenres.14786.r31864

**Published:** 2022-04-27

**Authors:** Peter Bower

**Affiliations:** 1 National Institute for Health Research (NIHR) Older People and Frailty Policy Research Unit, School of Health Sciences, Faculty of Biology, Medicine and Health, University of Manchester, Manchester, UK

Apologies for the delay.

 I feel that the author response was very clear and helpful and am happy to proceed with the article.

Is the study design appropriate for the research question?

Yes

Is the rationale for, and objectives of, the study clearly described?

Yes

Are sufficient details of the methods provided to allow replication by others?

Partly

Are the datasets clearly presented in a useable and accessible format?

Not applicable

Reviewer Expertise:

Health services research and research methodology

I confirm that I have read this submission and believe that I have an appropriate level of expertise to confirm that it is of an acceptable scientific standard.

# Peer review: Reviewer response for version 1

**DOI:** 10.21956/hrbopenres.14403.r30615

**Published:** 2021-12-16

**Authors:** Felipe Barreto Schuch

**Affiliations:** 1 Department of Sports Methods and Techniques, Federal University of Santa Maria, Santa Maria, Brazil

This study protocol is interesting, but there are some aspects that deserve some attention.

1. The introduction provides a good rationale for why this topic is interesting. However, the authors are not specifying the novelty of this study. There is a previous systematic review on the topic and the authors must justify this study considering the state of the art. See my article: Schuch, Felipe Barreto, *et al*. "Neurobiological effects of exercise on major depressive disorder: a systematic review." *Neuroscience & Biobehavioral Reviews* 61 (2016): 1-11.
2. The eligibility criteria are not informative enough. Are the authors including both studies evaluating a single bout of exercise (acute effects) and studies with trials of several weeks of intervention (chronic effects)? 
3. If they include studies evaluating the acute effects of exercise, do they need a control group of people with MDD who did not exercise?
4. Data synthesis: Does the author think using the raw mean difference to summarize the effects is appropriate? Cytokine dosage is not standardized, and different kits may have different ranges, sensitivities.
5. Data acquisition: are the authors planning to extract data (means and standard deviations) from figures if these values are not being presented on tables or in the body of the text? If so, how?

Is the study design appropriate for the research question?

Yes

Is the rationale for, and objectives of, the study clearly described?

Yes

Are sufficient details of the methods provided to allow replication by others?

No

Are the datasets clearly presented in a useable and accessible format?

Not applicable

Reviewer Expertise:

Exercise and mental health

I confirm that I have read this submission and believe that I have an appropriate level of expertise to confirm that it is of an acceptable scientific standard, however I have significant reservations, as outlined above.

## References

[1] : Neurobiological effects of exercise on major depressive disorder: A systematic review. *Neurosci Biobehav Rev* .2016;61: 10.1016/j.neubiorev.2015.11.012 1-11 10.1016/j.neubiorev.2015.11.012 26657969

# Response

**Published:** 2022-04-01

**Authors:** Jason Martin

**Affiliations:** University College Cork, Ireland

***1) The introduction provides a good rationale for why this topic is interesting. However, the authors are not specifying the novelty of this study. There is a previous systematic review on the topic and the authors must justify this study considering the state of the art. See my article: Schuch, Felipe Barreto, et al. "Neurobiological effects of exercise on major depressive disorder: a systematic review." Neuroscience & Biobehavioral Reviews 61 (2016): 1-11.***

 We thank the reviewer for this comment – We are aware of the authors excellent article detailing the state of the art up to 2016 of acute and chronic biological responses to exercise in people with MDD. We apologise for the oversight in omitting this citation from our review previously and have now included this article (page 5, paragraph 3) in our revised submission.

 We believe that a 6-year time gap is an appropriate timeframe within which to look anew on the data. The additional novelty of our review is its specific focus on how quantified exercise interventions modulate inflammatory biomarkers in MDD patients that have been published to date rather than the authors more expansive review that accounts the adaptations on neurogenesis, inflammation biomarkers and brain structure and how associations between improved depressive symptoms relate to hippocampal volume. We believe that our review will extend the observations around inflammation specifically beyond 2016 and is necessary to restate that the paucity of studies remains in this area of research and that limitations presented within existing studies precludes a definitive conclusion that exercise reduces inflammation in people with MDD.

***The eligibility criteria are not informative enough. Are the authors including both studies evaluating a single bout of exercise (acute effects) and studies with trials of several weeks of intervention (chronic effects)?*** 

 For clarity, within this review we aim to include all existing studies that evaluate the effect of a single bout of exercise or the effect of repeated exercise training over a longer time period (e.g., weeks - months, etc...). To help make this information clear and informative to the reader we now refer to chronic exercise (defined as repeated bouts of exercise) as simply "chronic" and acute exercise (defined as a single bout of exercise) as "acute". Importantly, the exercise interventions included must provide clear quantification of exercise intensity using a clearly specified unit of measure (as example: work rate specified in watts, joules, heart rate, or a percentage of maximal oxygen uptake or peak oxygen uptake) and with a clearly stated duration.

***2) If they include studies evaluating the acute effects of exercise, do they need a control group of people with MDD who did not exercise?***

  We agree that the inclusion of a control group of people with MDD is an important comparator group. The aim is to include those studies that evaluate the effects of an exercise-based intervention, be it a single bout or a longer-term training intervention, that investigate the effects of exercise on inflammation biomarkers, using controls that have been randomly allocated or created using another mechanism, but not before-and-after studies without controls. Ideally, these would be randomised control trials that used have used healthy controls, and/or depressed participants as controls. We intend in our discussion to comment on some inconsistencies in the existing literature on the definition of a control intervention. 

***3) Data synthesis: Does the author think using the raw mean difference to summarize the effects is appropriate? Cytokine dosage is not standardized, and different kits may have different ranges, sensitivities.***

 We thank the reviewer for raising this very good point and the lack of standardisation across the kits used to analyse cytokine levels will be an important discussion point. The standardized mean difference (SMD) test will be used to calculate the effect sizes of each mediator (see for example: Hedges and Olkin, 2014. Statistical Method for Meta-analysis. Academic Press.). For studies that evaluate the acute effects of exercise, the effect size will be calculated as the change from pre to post-test. The baseline or rest measure prior to exercise value will be used as the pre-test value. For post-test, the measure acquired immediately after exercise will be used. Where discrepancies/differences in sample timings exist, we will indicate this to allow the reader to make an informed interpretation of the observations reported.  

***4) Data acquisition: are the authors planning to extract data (means and standard deviations) from figures if these values are not being presented on tables or in the body of the text? If so, how?***

 Initially, we will contact manuscript authors to request the raw data or the reported data to avoid the need to extract data from figures, should the required data be available. Where no data is available, this will be identified and detailed with the review for transparency. Where data is required to be extracted from figures, due to no other source being forthcoming or available the following will be used. Medians and inter-quartile ranges will be transformed to means (M) and standard deviations (SD) using a web-based tool (Mean Variance Estimation, http://www.math.hkbu.edu.hk/~tongt/papers/median2mean.html). For data that are significantly skewed, an alternative validated procedure will be followed (Wan *et al*., 2014). Where possible and not otherwise available, numerical data will be extracted from graphs using WebPlotDigitizer (Rohatgi, 2020) and Adobe Acrobat's inbuilt measuring tool (Adobe Systems Inc., California, USA), as previously done by others (Safadi *et al*., 2021).

- *Wan X, Wang W, Liu J, et al. Estimating the sample mean and standard deviation from the sample size, median, range and/or interquartile range. BMC Med Res Methodol; 14. Epub ahead of print 19 December 2014. DOI: 10.1186/1471-2288-14-135.*
- *Rohatgi A. WebPlotDigitizer. California, USA, https://automeris.io/WebPlotDigitizer (2020).*
- *Safadi JM, Quinton AMG, Lennox BR, et al. Gut dysbiosis in severe mental illness and chronic fatigue: a novel trans-diagnostic construct? A systematic review and meta-analysis. Molecular Psychiatry 2021; 1–13. *

# Peer review: Reviewer response for version 1

**DOI:** 10.21956/hrbopenres.14403.r29777

**Published:** 2021-07-21

**Authors:** Peter Bower

**Affiliations:** 1 National Institute for Health Research (NIHR) Older People and Frailty Policy Research Unit, School of Health Sciences, Faculty of Biology, Medicine and Health, University of Manchester, Manchester, UK

Thank you for the opportunity to review this interesting protocol.

 I thought it was clear and well presented, but had some minor comments around terminology and clarity

 The purpose of this research is to ‘systematically review previous studies that have investigated the effects of quantified exercise interventions on inflammatory cytokine and biomarker levels in individuals diagnosed with MDD’. The statement ‘as compared to controls without any other major psychiatric or somatic co-morbidities’ is odd as it makes it sounds like these are selected on the basis of different criteria. Can they clarify the scope of ‘interventional’? I think that means with controls which can be randomly allocated or created using another mechanism, but not before-and-after studies without controls. Is that correct?

 I was a little unclear about the term ‘quantified exercise interventions’. I think the distinction they make with PA is clear and helpful, but what does ‘quantified’ mean? Does this mean that the exercise regime has to have a clearly stated duration and intensity? So the intervention needs to be quantified, even if adherence to the ideal is not perfect? How will they deal with adherence issues in the analysis? Or is this restricted to studies under near-laboratory conditions where adherence is high? This is a little out of my area of expertise so this could be usefully clarified.

 I felt that the statement about the review having the function to ‘inform areas for further research and identify gaps in the field’ could be clarified. What areas particularly?

 I always think it is odd to state that systematic reviews and meta-analyses analyses will be ‘excluded’. As is made clear later, they will be farmed for studies, not excluded in the same way as other studies.

 I am not sure of the meaning of the term ‘sub-depressive symptoms’? Do they mean sub-threshold depression?

 This is not my area at all, but are there quality criteria for assessment of the inflammatory cytokines and biomarkers?

 I did not really understand the phrase ‘The risk of confounds will be reduced by independently examining each study for potential confounds and how they were controlled for in the published study design (e.g., inclusion criteria selectivity) or analysis (e.g. employing adjustment techniques or analysis of covariance)’. How does this play out in the analysis?

 What is meant by ‘intervention type (i.e., acute or chronic)’? Those terms are not used in the description of interventions on page 4. Does that mean intervention FOR acute and chronic depression? I wasn’t sure those terms were current in psychiatry.

 They state that ‘the pooled OR and standard error (SE) will be used to generate a funnel plot in order to estimate the likelihood of publication bias if 10 or more studies are included in the meta-analysis’. Isn’t the main analysis using continuous measures? What are the ORs they are referring to?

 In limitations, they state ‘if possible, subgroup analyses will be undertaken to evaluate the impact of these sources of heterogeneity’. This seems to be something that should be in the methods.

Is the study design appropriate for the research question?

Yes

Is the rationale for, and objectives of, the study clearly described?

Yes

Are sufficient details of the methods provided to allow replication by others?

Partly

Are the datasets clearly presented in a useable and accessible format?

Not applicable

Reviewer Expertise:

Health services research and research methodology

I confirm that I have read this submission and believe that I have an appropriate level of expertise to confirm that it is of an acceptable scientific standard, however I have significant reservations, as outlined above.

# Response

**Published:** 2022-04-01

**Authors:** Jason Martin

**Affiliations:** University College Cork, Ireland

***The purpose of this research is to ‘systematically review previous studies that have investigated the effects of quantified exercise interventions on inflammatory cytokine and biomarker levels in individuals diagnosed with MDD’. The statement ‘as compared to controls without any other major psychiatric or somatic co-morbidities’ is odd as it makes it sounds like these are selected on the basis of different criteria. Can they clarify the scope of ‘interventional’? I think that means with controls which can be randomly allocated or created using another mechanism, but not before-and-after studies without controls. Is that correct?***

 Yes - we agree that the statement should be clarified. We have removed the statement ‘as compared to controls without any other major psychiatric or somatic co-morbidities’ so that the statement now reads.

 "The purpose of this research is to systematically review previous studies that have investigated the effects of quantified exercise interventions on inflammatory cytokine and biomarker levels in individuals diagnosed with MDD, via validated criteria."

 The reviewer is correct about the scope of ‘interventional’ as this does refer to studies that had the aim to evaluate the effects of an exercise-based intervention (a single bout or a training intervention) on inflammation biomarkers, using controls which have been randomly allocated or created using another mechanism, but not before-and-after studies without controls.  Ideally, these would be randomised control trials that used have used healthy controls, and/or depressed participants as controls – that said, we do not want to restrict this aspect as the existing literature appears to be inconsistent on the type of control participant used.

***I was a little unclear about the term ‘quantified exercise interventions’. I think the distinction they make with PA is clear and helpful, but what does ‘quantified’ mean? Does this mean that the exercise regime has to have a clearly stated duration and intensity? So the intervention needs to be quantified, even if adherence to the ideal is not perfect? How will they deal with adherence issues in the analysis? Or is this restricted to studies under near-laboratory conditions where adherence is high? This is a little out of my area of expertise so this could be usefully clarified.***

 Quantified exercise interventions means that the exercise intensity has been quantified using a clearly specified unit of measure (as example: work rate specified in watts, joules, heart rate, or a percentage of maximal oxygen uptake or peak oxygen uptake) and with a clearly stated duration.

 The issue of adherence is a great point to raise and will certainly be noted and discussed in detail. We intend to identify where and how studies have dealt with measuring adherence since we currently believe that some but not all studies will have been able to measure and/or report adherence.

***I felt that the statement about the review having the function to ‘inform areas for further research and identify gaps in the field’ could be clarified. What areas particularly?***

 We aim to inform future exercise-related research that investigates the topic of depression and inflammation by:

1. Providing a concise summary of the existing evidence thus far, the conclusions that can be made about the response of inflammatory biomarkers to exercise. To make clear the strengths and weaknesses of current research, to help focus future research to be able to have a better understanding of the mechanisms of action that underpin exercise interventions in stress-related disorders such as depression, and to hopefully, bring about some clarity about the optimal exercise intensity and/or duration required to have a beneficial intervention where possible.
2. Critique study designs to identify where improvements can be made.
3. Understand which biomarkers have been robustly investigated vs. those that have received little or no attention so that we are able to suggest avenues of exploration for future (e.g., su-PAR).

***I always think it is odd to state that systematic reviews and meta-analyses analyses will be ‘excluded’. As is made clear later, they will be farmed for studies, not excluded in the same way as other studies.***

 We have clarified this statement within the study design paragraph (Page 7, paragraph 1) to now read:

 "The following study designs will be excluded from this review and meta-analysis: case-studies, qualitative studies or quantitative studies performed on animal models or human populations with depressive symptoms but are not diagnosed with MDD or those with psychiatric or somatic co-morbidities. Systematic reviews and meta-analyses will be used a resource to identify relevant/eligible studies but will not be reported herein."

***I am not sure of the meaning of the term ‘sub-depressive symptoms’? Do they mean sub-threshold depression?***

 The term 'sub-depressive symptoms’ was used to refer to subclinical symptoms of depression that fall below the threshold to be clear categorised and diagnosable as major depressive disorder. This phrase has been changed to make it more clear "The following study designs will be excluded from review and meta-analysis: case-studies, qualitative studies or quantitative studies performed on animal models or human populations with: no clear MDD diagnosis; or with subclinical symptoms of depression; or those with psychiatric or somatic co-morbidities." (Page 7, paragraph 1)

***This is not my area at all, but are there quality criteria for assessment of the inflammatory cytokines and biomarkers?***

 There are a variety of research tools and experimental methods available and used for the assessment of inflammatory cytokines and other biomarkers, not all of which are certified as diagnostic in nature. Nevertheless, there are quality control and performance evaluation features which are reported by manufacturers for each assay type including for example with ELISA assays, linearity, precision, limits of detection, recovery and stability, and ROC curves.

 The assessment of biomarker validation and qualification is beyond the scope of this review.

 It is possible that authors may report information about the ROC curves as indicators of accuracy and performance indicators for analysis using ELISA (as example) to have understand the sensitivity and specificity, and the relevant optimal cut-off value used for ELISA-quantified biomarkers. Currently, it does not appear to be common practice to report such information in this research field - this may be in part due to the high performance (~>0.97) for an ELISA kit today.  

***I did not really understand the phrase ‘The risk of confounds will be reduced by independently examining each study for potential confounds and how they were controlled for in the published study design (e.g., inclusion criteria selectivity) or analysis (e.g., employing adjustment techniques or analysis of covariance)’. How does this play out in the analysis?***

 This was a phrase we used to try and encapsulate all the PRISMA guidelines in a simple phrase. On reflection, we agree this is not as clear as we would have liked. We have simplified this sentence to read "The risk of confounds will be explored and identified by in each study at the quality assessment stage by examining the published study design (e.g., inclusion criteria, selectivity) or analysis (e.g., employing adjustment techniques or analysis of covariance)." (Page 9-paragraph 5)

 This information will be collated and reported using the PRISMA flowchart, further a Cochrane risk of bias assessment will be performed and reported for all eligible studies.

***What is meant by ‘intervention type (i.e., acute or chronic)’? Those terms are not used in the description of interventions on page 4. Does that mean intervention FOR acute and chronic depression? I wasn’t sure those terms were current in psychiatry.***

 The term "acute" and "chronic" was meant to refer to the type of exercise training. We very much thank the reviewer for indicating that this is not very clear at present. We have attempted to make it clearer with the inclusion of an explanation:

 "A typical exercise training intervention requires an individual to repeat bouts of exercise over a short (1-2 weeks) or longer time period (i.e., weeks, months, or years) and can be defined as “chronic exercise” while, “acute exercise” is defined as a single bout. For clarity, within this review we now refer to chronic exercise (defined as repeated bouts of exercise) as simply "chronic" and acute exercise (defined as a single bout of exercise) as "acute."" (see objective 3 on Page 6 and Page 9-paragraph 1)

***They state that ‘the pooled OR and standard error (SE) will be used to generate a funnel plot in order to estimate the likelihood of publication bias if 10 or more studies are included in the meta-analysis’. Isn’t the main analysis using continuous measures? What are the ORs they are referring to?***

 We very much thank the reviewer for highlighting this typo we missed - this should have been SMDs and not OR here.  This has been corrected. The main analysis is using continuous measures.

***In limitations, they state ‘if possible, subgroup analyses will be undertaken to evaluate the impact of these sources of heterogeneity’. This seems to be something that should be in the methods**.* 

 We thank the reviewer for highlighting this point and have relocated this sentence to the data synthesis section.
